# A commentary on Eaves et al. with a special focus on clinical neurorehabilitation

**DOI:** 10.1007/s00426-023-01901-0

**Published:** 2023-12-11

**Authors:** Corina Schuster-Amft, Frank Behrendt

**Affiliations:** 1 Research Department, Reha Rheinfelden, Rheinfelden, Switzerland; 2 School of Engineering and Computer Science, Bern University of Applied Sciences, Biel, Switzerland; 3 Department of Sport, Physical Exercise and Health, University of Basel, Basel, Switzerland

## Abstract

We very much appreciate the theoretical foundations and considerations of AO, MI, and their combination AO + MI by Eaves et al. In their exploratory review, the authors highlight the beneficial effects of the combined use of AO and MI, with a particular focus on synchronous AO and MI. From a neurorehabilitation perspective, different processes may apply to patients, particularly after a stroke. As suggested by Eaves et al., the cognitive load might prevent the use of synchronous AO + MI and the asynchronous application of AO and MI might be indicated. Furthermore, some aspects should be considered when applying AO + MI in rehabilitation: screening for the patients’ cognitive capabilities and MI ability, and a familiarisation programme for AO and MI, before starting with an AO + MI training. With their review, Eaves et al. propose a number of research questions in the field of neurorehabilitation that urgently need to be addressed: the use of asynchronous vs. synchronous AOMI, observation and imagination with or without errors, or use of different MI perspectives and modes in different learning stages. This commentary provides some additional suggestions on patients’ MI ability and cognitive level, MI familiarisation and detailed reporting recommendations to transfer Eaves et al. findings into clinical practice.

In their recently published comprehensive and informative review, Eaves et al. (2022) highlight the beneficial effects of the combined use of action observation (AO) and motor imagery (MI) in healthy individuals or athletes and in rehabilitation. We very much appreciate the theoretical foundations and considerations with a particular focus on synchronous AO and MI in light of the current state of research in this field. Since previous reviews about MI during AO (Eaves et al., 2016; Vogt et al., 2013), there has been an encouraging increase in the number of publications on the various possible applications of the combination of the two methods. The current review also covers a variety of examples of how MI and AO can complement and interact with each other across different user groups: be they beginners of a motor skill to be acquired, advanced learners, or in the course of rehabilitation. The authors additionally review empirical evidence for the advantage of AO + MI, different forms of applications and models of AO + MI with regard to observing errors and mixed-skill models, the potential effectiveness of variable AO + MI scenarios, using AO as surrogate in MI in case a visual feedback is not available, factors moderating the effectiveness of AO + MI, the spectrum of AO + MI states and the differences and similarities in the underlying neurocognitive processes: the Dual Action Simulation hypothesis with regard to AO + MI. However, coming from a more clinical background, we would like to add some thoughts from that perspective.

An almost incidental mention, but a very important tool is indeed the glossary that Eaves and colleagues have compiled. It supports the correct and consistent use of terminology related to AO, MI and AOMI and aims to help clinicians more easily understand research findings and translate it into everyday practice. As part of our efforts in the latter, we also could find a positive effect of combined AOMI on motor learning in patients and healthy individuals in our own ongoing systematic review and meta-analysis. The studies analysed so far support a beneficial effect on the acquisition of motor skills, for example in children with Developmental Coordination Disorders (Marshall et al., 2020; Scott et al., 2019, 2020) and patients after a stroke (Binks et al., 2023). There is also first evidence for patients with Parkinson’s disease indicating that home-based AO + MI training using mobile technology is feasible and may have a beneficial effect (Bek et al., 2021).

Treatments in neurorehabilitation often involve a series of activities and exercises that the therapist presents to the patient one or more times before the patient performs them physically. In their Cochrane Review, Borges et al. (2018) found only a very small effect of pure AO on upper limb motor function that even did not persist after a 6 month follow-up period in stroke survivors. Here, real and video presentations were used as the AO method, with one or more trials in various combinations with physical performance, e.g. 1 min of observation combined with 2 min of physical performance for a total of 5–10 min. Indeed, it would be helpful to have some form of MI component available as an option for treatment to potentially enhance the effect. It is likely that some patients will spontaneously use MI during AO. However, MI instruction certainly has the potential to positively influence the therapy of those patients, who do not spontaneously use MI.

Certainly, as suggested by Eaves et al. (2022), it should be considered that the increased cognitive load may limit the use of synchronous AO + MI in certain user groups (Di Rienzo et al., 2019; Emerson et al., 2022) arguing for an asynchronous application of AO and MI in certain cases. Depending on MI ability and experience, it might moreover be beneficial to use AO alone or followed by MI in the first phase of rehabilitation, as it is less cognitively demanding (Emerson et al., 2018). Furthermore, age is certainly an important factor to consider in this context. (de Vries & Mulder, 2007) and (Gäumann et al., 2021) investigated age as a factor influencing MI ability and in both papers, the authors stated that with higher age (65 years or older), MI ability might decrease. Thus, in relation to age or as a consequence of brain injury, healthy older individuals and patients should be screened for cognitive decline. Further, from a neurorehabilitation perspective, assessment of MI ability is also essential before MI intervention begins. Recent evidence supports the benefits of assessing it in advance (Yasui et al., 2019). In our patient studies, we always apply a battery of MI ability assessments with our patients, e.g. the Body Rotation Task, Mental Chronometry, and a subjective MI questionnaire (Suica et al., 2022). Here, the subjective MI questionnaires already use AO as part of the evaluation process. To assess the different components of MI ability, such as generation, maintenance and manipulation, and to decide how to proceed with treatment, it is advisable to use multiple measures (Kraeutner et al., 2020).

There are two further important aspects to consider when using combined AO + MI: First, depending on the cognitive status, one or more familiarisation sessions might be conducted to familiarise healthy individuals or especially patients with the interventions (Wondrusch & Schuster-Amft, 2013). In our experience, this procedure can help patients learn about the interventions’ mechanisms of action, its effect, and how it could be used with or without supervision. Second, structured reporting of intervention methodology would facilitate the transition from research to clinical rehabilitation routine. Here, the suggested PETTLEP model (Holmes & Collins, 2001) or the overview of training elements by Schuster et al. (2011) could provide a reporting guide, e.g., position, location, perspective, mode, presenting angles. In addition, a detailed reporting would help to verify research findings. Possible tools to assess the cognitive status would be the Mini-Mental Status Exam (Folstein et al., 1975) and the Montreal Cognitive Assessment (Nasreddine et al., 2005).

Finally, Eaves and colleagues propose a portfolio of research questions in the field of neurorehabilitation that urgently need to be addressed: the use of asynchronous vs. synchronous AOMI, the observation and imagination with or without errors, or the use of different MI perspectives and modes in different learning stages. We look forward to the results of methodologically sound randomised controlled trials that address the above examples of AOMI in clinical neurorehabilitation, particularly in stroke rehabilitation. In our view, AO + MI has the potential to be part of routine treatment in neurorehabilitation, but it has not yet found its way into clinical practice. Screening of patients’ cognitive level, MI ability and familiarisation and, more importantly for reproducibility, structured reporting of all aspects of the AOMI sessions or training are strongly recommended.

## Data Availability

Not applicable.

## References

[CR1] Bek, J., Holmes, P. S., Craig, C. E., Franklin, Z. C., Sullivan, M., Webb, J., Crawford, T. J., Vogt, S., Gowen, E., & Poliakoff, E. (2021). Action imagery and observation in neurorehabilitation for parkinson’s disease (action-pd): Development of a user- informed home training intervention to improve functional hand movements. *Parkinson’s Disease*. 10.1155/2021/4559519 34336183 10.1155/2021/4559519 PMC8324342

[CR2] Binks, J. A., Emerson, J. R., Scott, M. W., Wilson, C., van Schaik, P., & Eaves, D. L. (2023). Enhancing upper-limb neurorehabilitation in chronic stroke survivors using combined action observation and motor imagery therapy. *Frontiers in Neurology*. 10.3389/fneur.2023.1097422 36937513 10.3389/fneur.2023.1097422 PMC10017546

[CR3] Borges, L. R., Fernandes, A. B., Melo, L. P., Guerra, R. O., & Campos, T. F. (2018). Action observation for upper limb rehabilitation after stroke. *The Cochrane Database of Systematic Reviews*. 10.1002/14651858.CD011887.pub2 30380586 10.1002/14651858.CD011887.pub2 PMC6517007

[CR4] de Vries, S., & Mulder, T. (2007). Motor imagery and stroke rehabilitation: A critical discussion. *Journal of Rehabilitation Medicine*. 10.2340/16501977-0020 17225031 10.2340/16501977-0020

[CR5] Di Rienzo, F., Joassy, P., Kanthack, T., MacIntyre, T. E., Debarnot, U., Blache, Y., Hautier, C., Collet, C., & Guillot, A. (2019). Effects of action observation and action observation combined with motor imagery on maximal isometric strength. *Neuroscience,**418*, 82–95. 10.1016/j.neuroscience.2019.08.025 31442568 10.1016/j.neuroscience.2019.08.025

[CR6] Eaves, D. L., Hodges, N. J., Buckingham, G., Buccino, G., & Vogt, S. (2022). Enhancing motor imagery practice using synchronous action observation. *Psychological Research Psychologische Forschung*. 10.1007/s00426-022-01768-7 36574019 10.1007/s00426-022-01768-7 PMC11315722

[CR7] Eaves, D. L., Riach, M., Holmes, P. S., & Wright, D. J. (2016). Motor imagery during action observation: A brief review of evidence, theory and future research opportunities. *Frontiers in Neuroscience*. 10.3389/fnins.2016.00514 27917103 10.3389/fnins.2016.00514 PMC5116576

[CR8] Emerson, J. R., Binks, J. A., Scott, M. W., Kenny, R. P. W., & Eaves, D. L. (2018). Combined action observation and motor imagery therapy: A novel method for post-stroke motor rehabilitation. *AIMS Neuroscience,**5*(4), 236–252. 10.3934/Neuroscience.2018.4.236 32341964 10.3934/Neuroscience.2018.4.236 PMC7179337

[CR9] Emerson, J. R., Scott, M. W., van Schaik, P., Butcher, N., Kenny, R. P. W., & Eaves, D. L. (2022). A neural signature for combined action observation and motor imagery? An fNIRS study into prefrontal activation, automatic imitation, and self–other perceptions. *Brain and Behavior*. 10.1002/brb3.2407 34994997 10.1002/brb3.2407 PMC8865155

[CR10] Folstein, M. F., Folstein, S. E., & McHugh, P. R. (1975). “Mini-mental state”. A practical method for grading the cognitive state of patients for the clinician. *Journal of Psychiatric Research,**12*(3), 189–198. 1202204 10.1016/0022-3956(75)90026-6

[CR11] Gäumann, S., Gerber, R. S., Suica, Z., Wandel, J., & Schuster-Amft, C. (2021). A different point of view: The evaluation of motor imagery perspectives in patients with sensorimotor impairments in a longitudinal study. *BMC Neurology,**21*(1), 297. 10.1186/s12883-021-02266-w 34315411 10.1186/s12883-021-02266-w PMC8314460

[CR12] Holmes, P. S., & Collins, D. J. (2001). The PETTLEP approach to motor imagery: A functional equivalence model for sport psychologists. *Journal of Applied Sport Psychology,**13*(1), 60–83. 10.1080/10413200109339004 10.1080/10413200109339004

[CR13] Kraeutner, S. N., Eppler, S. N., Stratas, A., & Boe, S. G. (2020). Generate, maintain, manipulate? Exploring the multidimensional nature of motor imagery. *Psychology of Sport and Exercise*. 10.1016/j.psychsport.2020.101673 10.1016/j.psychsport.2020.101673

[CR14] Marshall, B., Wright, D. J., Holmes, P. S., Williams, J., & Wood, G. (2020). Combined action observation and motor imagery facilitates visuomotor adaptation in children with developmental coordination disorder. *Research in Developmental Disabilities,**98*, 103570. 10.1016/j.ridd.2019.103570 31918039 10.1016/j.ridd.2019.103570

[CR15] Nasreddine, Z. S., Phillips, N. A., Bédirian, V., Charbonneau, S., Whitehead, V., Collin, I., Cummings, J. L., & Chertkow, H. (2005). The Montreal Cognitive Assessment, MoCA: A brief screening tool for mild cognitive impairment. *Journal of the American Geriatrics Society*. 10.1111/j.1532-5415.2005.53221.x 15817019 10.1111/j.1532-5415.2005.53221.x

[CR100] Schuster, C., Hilfiker, R., Amft, O., et al. (2011). Best practice for motor imagery: a systematic literature review on motor imagery training elements in five different disciplines. *BMC Medicine*, *9*, 75. 10.1186/1741-7015-9-75. 21682867 10.1186/1741-7015-9-75 PMC3141540

[CR16] Scott, M. W., Emerson, J. R., Dixon, J., Tayler, M. A., & Eaves, D. L. (2019). Motor imagery during action observation enhances automatic imitation in children with and without developmental coordination disorder. *Journal of Experimental Child Psychology,**183*, 242–260. 10.1016/j.jecp.2019.03.001 30921604 10.1016/j.jecp.2019.03.001

[CR17] Scott, M. W., Emerson, J. R., Dixon, J., Tayler, M. A., & Eaves, D. L. (2020). Motor imagery during action observation enhances imitation of everyday rhythmical actions in children with and without developmental coordination disorder. *Human Movement Science*. 10.1016/j.humov.2020.102620 32452437 10.1016/j.humov.2020.102620

[CR18] Suica, Z., Behrendt, F., Gäumann, S., Gerth, U., Schmidt-Trucksäss, A., Ettlin, T., & Schuster-Amft, C. (2022). Imagery ability assessments: A cross-disciplinary systematic review and quality evaluation of psychometric properties. *BMC Medicine,**20*(1), 166. 10.1186/s12916-022-02295-3 35491422 10.1186/s12916-022-02295-3 PMC9059408

[CR19] Vogt, S., Rienzo, F. D., Collet, C., Collins, A., & Guillot, A. (2013). Multiple roles of motor imagery during action observation. *Frontiers in Human Neuroscience*. 10.3389/fnhum.2013.00807 24324428 10.3389/fnhum.2013.00807 PMC3839009

[CR20] Wondrusch, C., & Schuster-Amft, C. (2013). A standardized motor imagery introduction program (MIIP) for neuro-rehabilitation: Development and evaluation. *Frontiers in Human Neuroscience,**7*, 477. 10.3389/fnhum.2013.00477 23986676 10.3389/fnhum.2013.00477 PMC3749428

[CR21] Yasui, T., Yamaguchi, T., Tanabe, S., Tatemoto, T., Takahashi, Y., Kondo, K., & Kawakami, M. (2019). Time course of changes in corticospinal excitability induced by motor imagery during action observation combined with peripheral nerve electrical stimulation. *Experimental Brain Research*. 10.1007/s00221-018-5454-5 30536148 10.1007/s00221-018-5454-5

